# Fast rule-based bioactivity prediction using associative classification mining

**DOI:** 10.1186/1758-2946-4-29

**Published:** 2012-11-23

**Authors:** Pulan Yu, David J Wild

**Affiliations:** 1 Indiana University School of Informatics and Computing, Bloomington, IN, 47408, USA

**Keywords:** Associative classification mining, Fingerprint, Pipeline Pilot, Bayesian, SVM

## Abstract

Relating chemical features to bioactivities is critical in molecular design and is used extensively in the lead discovery and optimization process. A variety of techniques from statistics, data mining and machine learning have been applied to this process. In this study, we utilize a collection of methods, called *associative classification mining* (*ACM*), which are popular in the data mining community, but so far have not been applied widely in cheminformatics. More specifically, classification based on predictive association rules (CPAR), classification based on multiple association rules (CMAR) and classification based on association rules (CBA) are employed on three datasets using various descriptor sets. Experimental evaluations on anti-tuberculosis (antiTB), mutagenicity and hERG (the human Ether-a-go-go-Related Gene) blocker datasets show that these three methods are computationally scalable and appropriate for high speed mining. Additionally, they provide comparable accuracy and efficiency to the commonly used Bayesian and support vector machines (SVM) methods, and produce highly interpretable models.

## Background

Classification is an essential part of data mining, and it involves predicting a categorical (discrete, unordered) label upon a set of attributes/variables. In cheminformatics, attributes usually are molecular descriptors such as structural fingerprints or physiochemical properties while the label represents bioactivity (for example, active/inactive class). Classification methods such as Decision forest [1], Bayesian classification [2-5], artificial neural networks(ANN), support vector machines (SVM) [6-8], k-nearest neighbor approach [9] and random forest [10]*inter alia* have been comprehensively used in cheminformatics, especially in drug discovery, to predict the activity of a compound based on its structural features. Several studies in the data mining community have shown that classification which is based on associations rule mining or so called *associative classification mining* (*ACM*) is able to build accurate classifiers [11-13] and is comparable to traditional methods such as decision trees, rule induction and probabilistic approaches. ACM is a data mining framework that employs association rule mining (ARM) methods to build classification systems, also known as associative classifiers. Recently, there have been some applications of ARM or ACM in the biological domain that are focused on genotype-phenotype mapping [14], gene expression data mining [15-17], protein-protein interaction (PPI) [18] or protein-DNA binding [19]. Genes found to be associated with each other by ARM or ACM can be helpful in building gene networks. Furthermore, the effect of cellular environment, drugs or other physiological conditions on gene expression can be uncovered by ACM as well [15]. In the cheminformatics field, there have been a few methods and typical applications using frequent itemset mining [20-23]. These methods enumerate fragments or the sub-graphs of the structure by applying sub-graph discovering algorithms on the topological structure of a molecule. Some [20] used an existing algorithm—frequent sub-graphs (FSG), while others [21,24] developed their own methods. Besides being used directly in associative classification, the mined frequent sub-graphs can be used as features for other methods such as SVM classifier [20]. However, to our best knowledge, compared with other fields, ACM has not been well explored.

**Figure 1:**
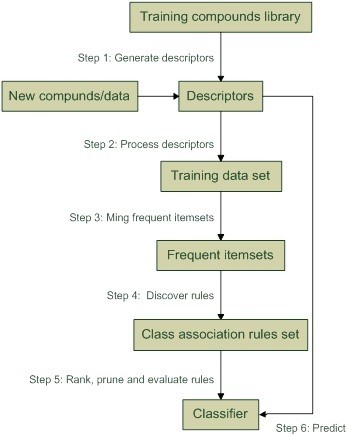
ACM framework.

ACM integrates association rule mining and classification. It utilizes a series of high quality class association rules (CARs) mined from the training dataset upon predefined minimum support and confidence constraints to build highly accurate classifiers [11]. Unlike most rule induction approaches which derive rules from part of the training library, ACM builds global classifiers based on the entire training data set. In recent years, a number of algorithms including classification based on association rules (CBA) [11], classification based on multiple association rules (CMAR) [12], classification based on predictive association rules (CPAR) [25], multi-class, multi-label associative classification (MMAC) [26], multi-class classification based on association rules (MCAR) [27], and generic association rules based classifier (GARC) [28] have been proposed. They all involve two basic steps: 1) generate classifiers consisting of a set of CARs; and 2) predicate new data by means of the classifier. The first step usually includes rule generation, rule ranking and rule pruning, and the second step involves rule selection, rule applying and classification. Figure 1 shows the framework we use in our study.

### Generation of classifiers

Let *A* be a set of m distinct attributes {A_1_, A_2_, …, A_i_} 1≤i≤m and C be a list of n distinct classes C_j_ 1≤j≤n. The attributes of A can be either continuous or categorical. For instance, the continuous ones can be pKa, solubility, or some quantum chemistry terms etc., and the categorical ones can be existence or nonexistence of certain features such as benzyl. The classes of C usually can be active or inactive, inhibiting or non-inhibiting. A training set T = (t_1_, t_2_, …, t_n_) is described as a set of transactions. Each transaction t_i_ is a combination of attribute values plus a class. For our case, a transaction is a chemical compound. For example, in Table 1, compound C1 is a transaction. *A* is a fingerprint set {Bit1, Bit2,…, Bit7} and C is a list consisting of “active” and “inactive”. Let *s* be a set of items with *s* ⊆ *A* ∪ *C*. *s* is referred to as an **itemset**. A **ruleitem** is an itemset which contains class information with an implication form of *X* → C, *X* ⊆ *A*. A possible ruleitem in Table 1 n=5 can be {Bit1 = 1, Bit7 = 0} → “Active” with support = 60% and confidence = 100%.

**Table 1:** A sample dataset with fingerprint as features

**Compound**	**Bit1**	**Bit2**	**Bit3**	**Bit4**	**Bit5**	**Bit6**	**Bit7**	**Class**
C1	**1**	1	0	0	1	0	**0**	Active
C2	0	0	1	0	0	1	**0**	Inactive
C3	**1**	1	0	1	0	0	1	Active
C4	**1**	0	1	1	0	0	**0**	Active
…	…	…	…	…	…	…	…	…
Cn	0	0	1	0	1	1	1	Inactive

Prior to rule generation, all **frequent ruleitems** are discovered. A ruleitem is **strong** if and only if it satisfies a minimum support θ (named minsup) threshold and minimum confidence δ (named minconf) threshold. The **support** of a ruleitem is the percentage of transactions in T that contain*X* ∪ *C* (i.e., the union of sets *X* and C, or say both *X* and C); the **confidence** of a ruleitem is the percentage of transactions in T having *X* that also contain C. Their probability definitions are support (*X* → C) = P (*X* ∪ *C*) and confidence (*X* → C) = P (C|*X*) respectively. For the above example, the support = 3/5 = 60% and confidence = 60%/60% = 100%. If δ = 10% and θ = 75%, then the example ruleitem is frequent and strong. Each ruleitem passing the minconf threshold is identified and a corresponding rule is generated. The derived rule from the example “if a compound’s fingerprint has Bit1 set and Bit7 not set then it tends to be active” provides intuitive interpretation of a relationship between the biological activity and chemical features.

Apriori [29], frequent pattern growth (FP-growth) [30] and Eclat [31] are the three most widely used basic algorithms of frequent itemset mining, which have been used for the first and major time consuming step. For example, CBA employs a traditional breadth-first method—Apriori [11], and CMAR utilizes the FP-growth approach [12]. Other algorithms are also applied, as an illustration, the modified first order inductive learner (FOIL) is adopted by CPAR [25]. Once all frequent rule items are discovered, they can be used for classifier generation and prediction. The size of the rule set is reduced in a process of pruning and evaluating with removing redundant and non-predictive rules to improve the efficiency and accuracy. The popular pruning techniques include chi-square testing, database coverage, rule redundancy, conflicting rules and pessimistic error estimation etc [13]. Pruning can be applied when extracting frequent ruleitems, generating rules (chi-square testing), or building classifiers (database coverage). Some pruning techniques such as database coverage and rule redundancy tend to produce small rule sets while others incline to generate relatively bigger classifiers. In practical usage, there is a trade-off between the size of classifiers and accuracies. After a classifier is built, it can be applied for next two steps: rule ranking and prediction.

### Prediction

Firstly, rules in the classifier are ranked by support, confidence and cardinality. In the event of a tie, most methods assign orders randomly, but Thabtah et al. argued that the class distribution frequency of the rule should be considered under this situation [13]. The prediction is based on either a single rule which matches the new data and has the highest precedence, or multiple rules that are all applicable to the new data. Different prediction methods are categorized as: maximum likelihood-based [11,27], score-based [12] and Laplace-based [25].

For some cases, the resulted classifier is more appealing than a “black box” such as ANN, SVM or Bayesian. Although most ACM algorithms have been tested against some standard data sets from UCI data collection; however, the application of these methods and interpretation of the generated ACM classifiers in terms of chemical features and bioactivity are not available.

In this paper we present data supporting the viewpoint that ACM can be used for modeling chemical datasets while preserving some appealing features from other methods.

## Experimental

### Datasets

(1) The hERG dataset is downloaded from pharmacoKinetics Knowledge Base (PKKB) [32]. The dataset contains 806 molecules with hERG activities. 495 compounds are from Li’s dataset [33] and 66 from WOMBAT-PK [34] database; the other 245 compounds are collected by PKKB from publications. Compounds are classified into blockers (IC50 less than or equal to 40μm) and non-blockers (IC50 greater than 40μm).

(2) The antituberculosis (antiTB) dataset is obtained from Prathipati’s paper [2]. According to this paper, the dataset contains a large number of curated and diverse chemical compounds which are appropriate for modeling. In this study, all 3,779 compounds are used. The compounds are classified into active and inactive groups using the same criterion as used in the paper— minimum inhibitory concentration (MIC) less than 5μM.

(3) The mutangenicity dataset contains 4,337 compounds with Ames test data and 2-D structures. The dataset is constructed from the available Ames test data by using the following criteria: a) standard Ames test data of *Salmonella Typhimurium* strains required for regulatory evaluation of drug approval; b) Ames test performed with standard plate method or preincubation method, either with or without a metabolic activation mixture. Compounds which contain at least one positive Ames test result are classified as mutagen, otherwise as non-mutagen [35].

These three datasets are characterized by their diversities ranging from 0.90-0.93 and the ratio of the number of compounds is hERG:antiTB:Mutagenicity=1:4.7:5.4 (Table 2). The diversity ensures multiple patterns, and the different sizes of the dataset can be used to investigate the relationship between performance and size.

**Table 2:** The characteristics of the data sets used in this paper

**Data set**	**hERG**	**antiTB**	**Mutagenicity**
Source	PKKB [32]	Prathipati et al. [2]	Jeroen et al. [35]
#Compounds	806	3,779	4,337
Diversity	0.90	0.90	0.93
Class	blocker/non-blocker	active/inactive	mutagen/non-mutagen

**Note**: The diversity of each dataset is the average distance of all molecules and is calculated based on ECFP_6 by using Pipeline Pilot. The distance is defined as (1- similarity) for every pair of molecules based on the specified fingerprint.

### Molecular Descriptors

In all experiments, the MDL public keys and PubChem’s CACTVS [36] are used for model development since they tend to yield high quality models [10,37,38]. Both fingerprints belong to structural fingerprints which encode a bit string based on the topological structure. The MDL public keys is generated by Pipeline Pilot [39]; the PubChem chemical fingerprint is produced by using an in-house program based on the Chemistry Development Kit (CDK) [40]. In addition to the above fingerprints, properties such as ADMET properties, physiochemical properties and simple counts of molecular features (Table 3) are included for model building as well.

**Table 3:** Property descriptors used in the modeling

**ADMET**	**ADMET_BBB_Level,ADMET_Absorption_Level,ADMET_CYP2D6,ADMET_PPB_Level**
**Physiochemical**	ALogP,Molecular_Solubility,Molecular_SurfaceArea,Molecular_PolarSurfaceArea,Molecular_FractionalPolarSurfaceArea,Molecular_SASA,Molecular_PolarSASA,Molecular_FractionalPolarSASA,Molecular_SAVol,ChemAxon_LogP,ChemAxon_Polarizability,ChemAxon_Refractivity,ChemAxon_TPSA,FormalCharge
**Simple counts**	Num_Atoms,Num_Bonds,Num_Hydrogens,Num_NegativeAtoms,Num_RingBonds,Num_RotatableBonds,Num_BridgeBonds,Num_Rings,Num_RingAssemblies,Num_Chains,Num_ChainAssemblies,Molecular_Weight,Num_H_Acceptors,Num_H_Donors,ChemAxon_HBA,ChemAxon_HBD

**Note**: All property descriptors are computed by using Pipeline Pilot. The name and meaning of property descriptors can be found in Pipeline Pilot help documents. In most cases, the meaning of a name can be determined from the name itself. For example, ADMET_BBB_LEVEL means ranking of the LogBB values by using Accelrys blood–brain barrier penetration model: 0 is very high; 1 is high; 2 is medium; 3 is low and 4 is undefined, namely, molecule is outside of the confidence area of the regression model used to calculate LogBB.

#### Properties

Both Naïve Bayesian (Bayesian) and ACM prefer categorical attributes since the conditional probability for Bayesian can be described using a smaller table and the number of itemsets for ACM can be significantly reduced. Meanwhile, converting continuous attributes into categorical attributes also helps treat all the attributes and the class identically. The quantitative/numeric attributes such as AlogP, molecular weight, number of H-acceptor, H-donor and rotation bonds are discretized into levels and the levels are mapped into categorical values. To demonstrate, for AlogP, we set 1 for 0≤AlogP≤3.5, 2 for 3.5<AlogP≤7 and 3 for 7<AlogP. For every data set, the entropy based methods are utilized for discretizing all the attributes, which has been done by Rapid miner 5.1 [41]. The process is performed by using the “Discretize by Entropy” operator in RapidMiner with default settings. Previous studies have shown that the performance of Bayesian algorithm can be significantly improved if entropy-based discretization is adopted [42,43]. As a result, all the continuous attributes are converted into categorical attributes for mining.

#### Fingerprints

Both MDL public keys and PubChem fingerprints are bit strings of fixed length with size of 166 and 881 respectively. There is a one-to-one mapping between the bits and molecule features ideal for our mining and model interpretation. Each bit can be set to 1 or 0 representing the existence or nonexistence of a predefined chemical feature. The bit string can be mined directly by the software package used in this research.

### Classification

All the computations are carried out on a PC Q6600 2.4GHz with 6G memory running on the 64-bit Windows 7 operating system. Results of the Bayesian and SVM are used as references. The computation and modeling of Bayesian and SVM are performed by using RapidMiner with default settings. As to speed, Bayesian is the fastest one; ACM is faster than SVM in most cases. For example, the computation time for mutagenicity dataset are 5 seconds, 20.5 minutes, 1.5 minutes, 5.7 minutes and 6.3 minutes for Bayesian, SVM, CPAR (there is another implementation which only takes 12 seconds), CMAR and CBA respectively.

## Methods

### Associative classification

The implementation of CBA, CPAR and CMAR (Table 4) from Coenen F [44]. are used in this study. We added functions such as outputting classifiers, calculating F_1_ scores and a graphical user interface (GUI). Parameters δ, min_gain, α and k are set to 0.55, 0.7, 2/3 and 5 respectively for CPAR. For CBA and CMAR computation, MinSup and MinConf are set as 1% and 50%. CBA v2.1 from Liu et al. [45] is used for rule formatting and feature analysis.

**Table 4:** Summary of used ACM methods

**Method**	**Summary**
**CBA**	Classification based on association rules [11,45] first discovers all rules by using Apriori approach, and then prunes rules by database coverage technique.
**CPAR**	Classification based on predictive association rules [25] uses a greedy approach—a weighted version of FOIL-gain to identify features and discover rules. A PNArray data structure is utilized to reduce storage space and computation time [13].
**CMAR**	Classification based on multiple association rules [12] employs FP-growth method to discover rules. FP-growth builds a FP-tree based on the dataset using less storage space and improves the efficiency of retrieving rules.

### Model Assessment and Evaluation

For all data sets, the classification performance is assessed by using 10-fold cross validation (CV). This method provides more reliable assessment of classifiers which generalizes well to new data. The accuracy of the classification can be determined by many existing evaluation methods such as error-rate, recall-precision, any label and label-weight etc. In this paper, we use F-score (F_1_ score or F-measure) to measure the overall performance.

(1) $$F_{1}=2*\frac{Precision*Recall}{Precision+Recall}$$

(2) $$Precision=\frac{true\;positive}{true\;positive+false\;positive}$$

(3) $$recall=\frac{true\;positive}{true\;positive+false\;negative}$$

To further study the robustness of the generated models, Y-randomization is applied to the antiTB dataset as an alternative validation method. Paola [46] recommended that Y-randomization and CV should be carried out in parallel to test the significance of the derived models. In this method, the bioactivity vector is randomly shuffled and a new model is generated based on the original feature matrix. The process is repeated five times and the resultant models are compared with the original one.

## Results and discussion

### Discretization

**Figure 2:**
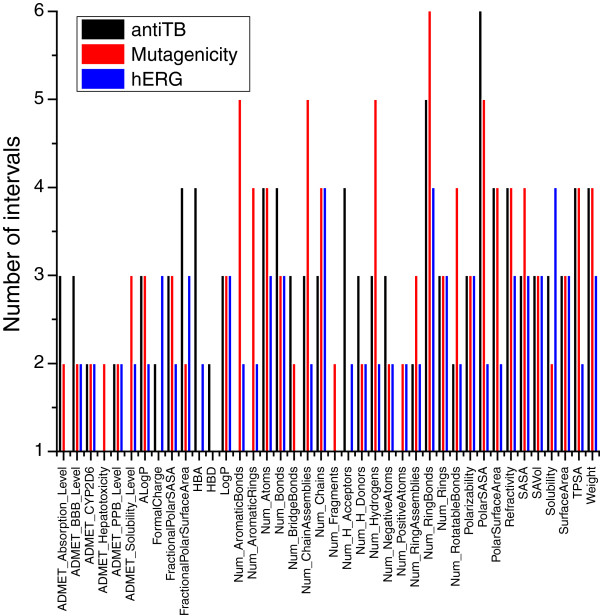
Discretization results of the antiTB datasets.

Figure 2 shows that all the properties of antiTB are discretized into levels from 2 (binary) to 6. For each property, the number of levels indicates how many splits are required to maximize the information gain. The type of properties, the number of compounds, the diversity of the dataset and the distribution of classes affect the discretization result. For instance, given the same property, the number of chains is split into different intervals proportionally according to the dataset. The entropy-based discretization process automatically removes the attribute with only one level. In fact, those attributes are not discretized since the entropy criterion is not met. Hence, although the same attributes are used for each dataset, the final attributes used for modeling are different. As an example, Num_AromaticBonds only exists in the mutagenicity and hERG datasets, while ADMET_Absorption_Level only exists in the antiTB and mutagenicity datasets.

### Effects of fingerprint size and encoding

As shown in Table 5, the fingerprint size and encoding scheme have a great impact on the effectiveness of the model. In our experiment, a larger fingerprint does not always afford a higher accuracy for all the approaches. The performance of fingerprints depends on the dataset and the methods used. To illustrate, for the mutagenicity dataset, the accuracy of MDL is higher than that of PubChem no matter what method is used. However, for the hERG dataset, the accuracy of PubChem is relatively higher when modeled by ACM; for the antiTB dataset, PubChem perform better for the SVM and Bayesian approaches. During the optimization of MDL keys, Durant et al. got similar results by comparing fingerprints with different sizes [47].

**Table 5:** **F**-**score of all the data sets using different descriptors or fingerprints**

**Data set**	**Descriptors**	**Classification Model**				
		**SVM**	**Bayesian**	**CPAR**	**CBA**	**CMAR**
**AntiTB**	**MDL**	61.82±2.96	63.00±2.97	70.78±3.51	**72**.**06**±**2**.**38**	69.84±2.14
	**Properties**	69.50±1.84	**70**.**48**±**1**.**80**	63.27±3.10	66.09±5.66	63.49±2.51
	**PubChem**	71.08±1.72	68.93±2.23	**74**.**15**±**1**.**75**	63.62±2.35	67.25±1.52
**Mutagenicity**	**MDL**	74.26±2.87	69.07±3.49	77.37±5.25	**77**.**75**±**4**.**89**	75.48±5.11
	**Properties**	70.04±3.99	68.82±6.13	66.75±2.74	**75**.**87**±**3**.**58**	74.57±5.44
	**PubChem**	72.67±3.80	66.41±3.66	75.77±4.16	**76**.**13**±**3**.**91**	71.91±5.38
**hERG**	**MDL**	62.62±6.73	70.08±9.64	72.75±12.26	69.20±6.84	**73**.**69**±**9**.**67**
	**Properties**	**80**.**82**±**7**.**22**	75.73±13.35	72.78±10.39	79.65±6.37	80.73±8.18
	**PubChem**	60.13±9.98	73.18±11.89	77.72±9.70	74.77±8.28	**78**.**03**±**9**.**79**

### Comparison of different approaches

Five models are built for each combination of dataset and feature type (e.g. for antiTB dataset when using MDL, antiTB_MDL will be used to represent one combination). In total, there are nine combinations of datasets and feature types which generate forty-five models. Table 5 shows that the overall F-score of ACM is comparable to or better than that of Bayesian and SVM. The highest F-score in each combination is shown in bold. Among the total nine combinations, only two are achieved by SVM and Bayesian which is 70.48±1.80 for the antiTB_MDL combination (Bayesian) and 80.80±7.22 for the hERG_properties (SVM). A simple ranking method can be used to compare CPAR, CBA and CMAR without considering the complexity of the classifier. For any scenario, the three approaches are assigned 1, 2 and 3 according to the accuracy with 1 for the most accurate. For example, for antiTB_MDL, CPAR is 2, CBA is 1 and CMAR is 3. The final rank is the average of all cases. The result is 2.11, 1.78 and 2.11 for CPAR, CBA and CMAR respectively, which shows the order of the accuracy is CBA > CPAR = CMAR in this study.

### Y-randomization

Table 6 shows the robustness of the models by the Y-randomization method. All randomized models perform worse than the original ones (bold) in terms of accuracy. The randomized rules generated also have low classification abilities on the original dataset. For example, a rule with confidence 98.82% in the randomized dataset has a confidence value of only 53% in the original dataset. This proves that models for the original dataset cannot be generated from the randomized datasets and also indicates that the good performance of the original models is not achieved by chance correlation or by structural redundancy of the datasets.

**Table 6:** **Accuracy of Y**-**randomization on antiTB**_ **MDL**

**Model**	**CPAR**	**CBA**	**CMAR**
**original**	**70**.**78**±**3**.**51**	**72**.**06**±**2**.**38**	**69**.**84**±**2**.**14**
**1**	44.25±19.38	43.08±3.97	44.03±4.68
**2**	40.35±19.54	49.04±2.92	51.00±2.81
**3**	39.27±11.29	48.98±3.94	45.77±3.61
**4**	57.83±8.73	50.66±2.37	48.24±3.84
**5**	57.85±6.11	52.62±3.11	51.05±5.45

### ACM classifiers and their chemical significance

27 ACM models are built in total in our study. For the classifier and significance analysis, CBA models for the antiTB dataset are chosen to demonstrate the analyzing strategies and their chemical significances. The same strategies can be applied to any other models and similar results can be obtained.

#### Single feature analysis

Some classifiers have around twenty rules and others may have several hundreds. The number of generated rules varies depending on several factors: the size of the dataset, features, algorithms etc. The results show that CMAR produces the biggest classifiers in most cases. Parameters can be tuned to reduce the size of the classifier but the accuracy may be lowered correspondingly. Another important character of the classifiers is the length of the rules, namely, the size of the ruleitem. In our study, the item size of CPAR ranges from one to seven. Although to reduce the total number of itemsets, the maximum length of CBA and CMAR is set to four, the length of the generated rules is mostly two or three. Longer rules sometimes can provide us more information about the compounds since they contain more structural fragments.

To analyze the importance of each feature to the activity, both the confidence and support are taken into account for each feature in each rule. A number R is assigned to each feature in the rule.

(4) $$R=Round\left(\frac{sign\left(existence\right)*\left(Support+Confidence\right)}{2}*100\right)$$

If a feature is existing, then sign = 1; otherwise sign = −1. The rank of this feature is the sum of R. With antiTB dataset as an example, Additional file 1: Table S1 shows the rank of each feature for active and inactive compounds respectively. Of particular interest are the features (yellow features in Additional file 1: Table S1) that exist only in active compounds and those only found in inactive compounds (red). For green features, their contributions to the bioactivity depend on other features that are in association with them. The MDL feature space is reduced from 166 to 101 for the antiTB dataset. The same analysis can be carried out for the PubChem fingerprint. To be noticed, the feature space for PubChem is remarkably reduced from 881 to 146. Although MDL and Pubchem use substantially different encoding schemes, the mined features are related, such as MDL 110 with PubChem 366, 117, 123 and 95, MDL 75 with PubChem 392 and MDL 22 with PubChem 116 (Additional file 1: Table S2 and S3). Among the top ten features, multiple features (Additional file 1: Table S4) are linked to each other.

**Figure 3:**
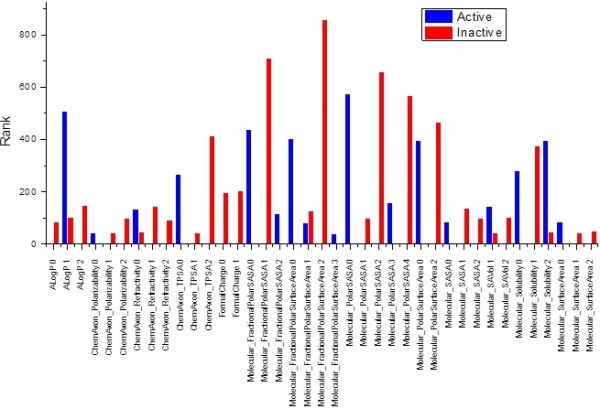
Rank of the levels of the properties for the antiTB dataset.

The property models can be analyzed in the same fashion. As mentioned above, properties are discretized into different levels. The physiochemical properties (Figure 3) suggest that the activity is closely related to AlogP, polar surface area and solvent accessible surface area since 0.985<AlogP 1<4.446, ChemAxon_TPSA 0 < 46.17, Molecular_PolarSASA 0< 74.521 and Molecular_PolarSurfaceArea 0< 47.92. Ronald’s model demonstrated that the best AlogP is from −2.165 to 1.373 [49] which overlaps a lot with our results. However, his model favors a relatively bigger PSA ranging from 55.121 to 94.036. A bigger PSA value sometimes will inhibit the intestinal absorption of an orally administrated drug.

#### Association rule analysis

The single feature analysis provides a lookup table (Additional file 1: Tables S1, S2, S3, and S4 and Figure 3) for general references of “good” or “bad” features. However, the information is not as complete as what the rules can provide. The discovered rules in this study represent a number of non-random and interesting relationships that can help rational molecular design and can ultimately be helpful for drug discovery. Depending on the implementations, the classifier may use up to *k* rules to determine the class of a compound where *k* may range from one to seven. The interpretation of the rules is straightforward given the meaning of each bit in the fingerprint or the names of the properties. For example the rule 1 in Table 7 states that if a compound contains substructure NCO (Bit 110) and A!O!A (Bit 126) then it is active. There are 23.10% percent of the compounds in the dataset that meet this requirement and among them 75.14% percent are active compounds. Rule 1–4 and their matched molecule examples are provided in Additional file 1: Table S5. Based on the meaning of each bit and SMARTS pattern, NCO is interpreted as N, C and O connect to each other by any bond type, and A!O!A as O connects to any atoms with non-ring bond. Compared with the top features mined by ECFP fingerprint [2], the G14 contains both NCO and A!O!A. In addition, rule 3 indicates that NCO and *~*(~*)(~*)~* which means any atom connects to 4 atoms with any bond type are also “good” features. This also matches the top features of G2, G3, G5, G6, G8, G9, and G10 in the same paper. Both rule analysis and single feature analysis imply NCO is a very important feature which is good for “active” and bad for “inactive”. Interestingly, a CoMFA study by Rahul [50] shows that the NCO group plays an important role in the pharmacophore model too.

**Table 7:** Selected association rules for the antiTB dataset

**Association rules**	**Support**	**Confidence**
MDL		
1	[#7]~[#6]~[#8] AND *!@[#8]!@* → class = active	23.10% 75.14%
2	Not [#7]~*~*~[#8] AND not [#7]!:*:* → class = inactive	21.38% 75. 50%
3	[#7]~[#6]~[#8] AND *~*(~*)(~*)~* → class = active	18.95% 81.98%
4	[#7]~*~[CH2]~* AND [#8]~[#6]~[#8] → class = active	18.37% 76.80%
Property		
5	ALogP[0.985 - 4.446] AND Num_RingBonds[>19] AND ADMET_CYP2D6[=0 ] → class = active	9.55% 74.64%
6	Num_Hydrogens[18–50] AND Molecular_Solubility[−12.036 - -7.198] AND Molecular_SASA[690.864 - 1058.920] → class = inactive	9.03% 78.31%
7	Molecular_FractionalPolarSASA[0.140 - 0.312] AND Molecular_Solubility[−12.036 - -7.198] AND ChemAxon_HBD[>3] → class = inactive	9.00% 91.84%
8	Num_Bonds[<30] AND ChemAxon_TPSA[<46.170] AND Molecular_FractionalPolarSASA[<0.140] → class = active	9.00% 81.57%

The property rules utilize a set of property levels to achieve relatively higher classification accuracy. Rule 5 employs ALogP with Num_RingBonds and non CYP2D6 inhibitor together to identify active compounds. Our previous single feature analysis discovered that an optimum ALogP was important for activity. The specific mechanism behinds the association of CYP2D6 level and antiTB activity is not clear. Several popular antiTB drugs such as isoniazid and rifampicin, induce certain CYP activity. A possible explanation of non CYP2D6 inhibitor related to active antiTB activity might be that some drugs are administrated as prodrug. Their active ingredients are metabolites depending on the CYP activity such as the undergoing drug SQ109 [51]. Finally, the level number of ring bonds can help researchers limit the number and size of the rings at the same time.

## Conclusions

ACM is a powerful tool for modeling as it not only offers comparable accuracy but also interpretability. In particular, the measures of descriptor importance can provide guidance for molecule design. It does not need prior feature selection or parameter tuning but preserves the most appealing feature of Bayesian and Decision Tress—the ability to handle a large number of descriptors simultaneously. Compared with some tree-based methods, models generated by ACM are relatively stable and their accuracies are higher. Therefore, the interpretability of the model is more reliable—an obvious advantage in contrast with “black-box” methods. The mined association rules represent the possible relationships between the structure and bioactivity. More functional rules can be found by using different features or criteria. Among the three methods studied, CBA has relative higher accuracy than CPAR and CMAR, and CMAR generates the biggest classifiers. Additionally, the classifier of CPAR has the longest rules.

Single feature analysis provides a fast way to access the “good” or “bad” features for antiTB compounds. The list of fingerprint bits preferentially presented in active or inactive compounds can be used as a guide for screening and optimizing. Depending on the attributes and the methods of discretization, both general and specific interpretations can be made from the ACM classifiers by combining chemical or biological knowledge. In each case the generated model indicates that a very strong relationship between the structural features and bioactivities exists in the studied datasets.

All ACM methods used here are called *traditional ACM* methods because they do not distinguish the difference of significance of features. For some cases, features are not equally important. For example, in our study, even though we know AlogP, ADMET_BBB_Level or Molecular_SASA are more important than others, traditional ACM is not able to incorporate this information during mining. Our next step will incorporate weight information of the features into ACM—weighted ACM, which can generate more correlated and important patterns [52-54]. Recently, knowledge from semantic ontologies is used to understand or interpret the meaning of the patterns produced by ACM [55]. Additionally, it is integrated into an existing rule reduction process to build concise, high quality and easily interpretable rule set [56]. At present, most of the ontology-driven mining in the biomedical domain uses the UMLS [57] or GO [58] ontology, but now several chemical information ontologies such as ChEBI [59] and CHEMINF [60] are available too. Our future work will try to improve current models by incorporating those ontologies constraints during the rule generation process. We envision that there will be more applications of ACM in the chemical domain.

## Competing interests

The authors declare that they have no competing interests.

## Authors' contribution

PLY and DJW conceived the study, PLY carried out the implementation, PLY and DJW wrote the manuscript. All contributed to the intellectual evolution of this project. Both authors have read and approved the final manuscript.

## Supplementary Material

Additional file 1 **Table S1.** MDL and PubChem feature rank in active and inactive compounds for antiTB. Note: This table is based on the antiTB dataset. If a feature exists (e.g. bit137=1), then sign = 1, otherwise (bit 137=0) sign = −1. Rank in Active means the rank of a feature in active compounds and Rank in Inactive for a feature in inactive compounds. The rank value is computed by equation 1. For Bit 137, it means both bit137=1 and bit137=0 are discovered in the rules for inactives. The rank for bit137=1 and bit137=0 for inactives is 44 and 83 respectively. Yellow features only exist in active compounds; red only in inactive compounds; green in both types. **Table S2:** Important MDL features for the antiTB dataset. Note: Each bit corresponds to a SMARTS pattern [48] which consists of two fundamental types of symbols: atoms and bonds. “*” means any atom, “A” an aliphatic atom, “~” any bond and “:” aromatic bond. So Bit 89, [#8]~*~*~*~[#8], means “two oxygen atoms connected by three unspecified atoms with any type of bonds”. **Table S3:** Important PubChem features for the antiTB dataset. **Table S4:** Related features among top 10 of MDL and PubChem fingerprints. Note: All visualized SMARTS patterns are generated by using smartsviewer from http://smartsview.zbh.uni-hamburg.de/. The color scheme uses the popular CPK coloring with green for fluorine, red for oxygen, black for carbon, yellow for sulfur and blue for nitrogen. **Table S5:** The matched molecules for rule 1–4 in Additional file 1: Table S3. Note: a. red shape is *!@[#8]!@* and green shape [#7]~[#6]~[#8] b. molecule does not contain the two substructures c. red shape is *~*(~*)(~*)~* and green shape is [#7]~[#6]~[#8] d. red shape is [#7]~*~[CH2]~* and green shape is [#8]~[#6]~[#8]. Click here for file
