# Supplementary material for: Fast rule-based bioactivity prediction using associative classification mining
Source: J Cheminform. 2012 Nov 23;4:29. doi: 10.1186/1758-2946-4-29 (PMC3515428; doi:10.1186/1758-2946-4-29)
Supplement: Additional file 1 — Table S1. MDL and PubChem feature rank in active and inactive compounds for antiTB. Note: This table is based on the antiTB dataset. If a feature exists (e.g. bit137=1), then sign = 1, otherwise (bit 137=0) sign = −1. Rank in Active means the rank of a feature in active compounds and Rank in Inactive for a feature in inactive compounds. The rank value is computed by equation 1. For Bit 137, it means both bit137=1 and bit137=0 are discovered in the rules for inactives. The rank for bit137=1 and bit137=0 for inactives is 44 and 83 respectively. Yellow features only exist in active compounds; red only in inactive compounds; green in both types. Table S2: Important MDL features for the antiTB dataset. Note: Each bit corresponds to a SMARTS pattern [48] which consists of two fundamental types of symbols: atoms and bonds. “*” means any atom, “A” an aliphatic atom, “~” any bond and “:” aromatic bond. So Bit 89, [#8]~*~*~*~[#8], means “two oxygen atoms connected by three unspecified atoms with any type of bonds”. Table S3: Important PubChem features for the antiTB dataset. Table S4: Related features among top 10 of MDL and PubChem fingerprints. Note: All visualized SMARTS patterns are generated by using smartsviewer from http://smartsview.zbh.uni-hamburg.de/. The color scheme uses the popular CPK coloring with green for fluorine, red for oxygen, black for carbon, yellow for sulfur and blue for nitrogen. Table S5: The matched molecules for rule 1–4 in Additional file 1: Table S3. Note: a. red shape is *!@[#8]!@* and green shape [#7]~[#6]~[#8] b. molecule does not contain the two substructures c. red shape is *~*(~*)(~*)~* and green shape is [#7]~[#6]~[#8] d. red shape is [#7]~*~[CH2]~* and green shape is [#8]~[#6]~[#8]. [file 1758-2946-4-29-S1.docx]

**Table S1** **MDL and PubChem feature rank in active and inactive compounds for antiTB**

| **MDL** | | | | | | **PubChem** | | | | | | | |
| --- | --- | --- | --- | --- | --- | --- | --- | --- | --- | --- | --- | --- | --- |
| **Bit** | **Rank in Actives** | | **Bit** | **Rank in Inactives** | | **Bit** | **Rank in Actives** | | **Bit** | **Rank in Inactives** | | **Bit** | **Rank Inactives** |
| 66 | 568 |  | 36 | 217 |  | 183 | 391 |  | 201 | 300 |  | 496 | -42 |
| 86 | 476 |  | 89 | 169 |  | 364 | 391 |  | 360 | 221 |  | 629 | -43 |
| 42 | 404 |  | 46 | 165 |  | 207 | 285 |  | 476 | 163 |  | 692 | -43 |
| 53 | 341 |  | 99 | 134 |  | 588 | 249 |  | 150 | 137 |  | 465 | -43 |
| 22 | 306 |  | 13 | 127 |  | 683 | 235 |  | 149 | 135 |  | 546 | -43 |
| 26 | 294 |  | 22 | 93 |  | 116 | 200 |  | 498 | 134 |  | 438 | -44 |
| 101 | 286 |  | 41 | 91 |  | 589 | 183 |  | 555 | 125 |  | 392 | -44 |
| 92 | 242 |  | 67 | 88 |  | 616 | 102 |  | 543 | 125 |  | 699 | -44 |
| 113 | 237 |  | 34 | 85 |  | 117 | 101 |  | 400 | 123 |  | 792 | -44 |
| 139 | 236 |  | 60 | 45 |  | 147 | 101 |  | 534 | 120 |  | 541 | -45 |
| 50 | 199 |  | 148 | 45 |  | 703 | 98 |  | 478 | 118 |  | 622 | -45 |
| 112 | 196 |  | 144 | 44 |  | 707 | 96 |  | 207 | 88 |  | 450 | -46 |
| 95 | 189 |  | 113 | 44 |  | 544 | 95 |  | 116 | 87 |  | 347 | -46 |
| 115 | 185 |  | 137 | 44 | -83 | 14 | 93 |  | 331 | 84 |  | 539 | -46 |
| 38 | 185 |  | 51 | 44 |  | 185 | 92 |  | 329 | 83 |  | 12 | -79 |
| 89 | 184 |  | 69 | 44 |  | 184 | 92 |  | 130 | 81 |  | 452 | -79 |
| 78 | 176 |  | 153 | 43 | -46 | 591 | 89 |  | 423 | 79 |  | 644 | -84 |
| 37 | 150 |  | 47 | 43 |  | 646 | 87 |  | 493 | 78 |  | 421 | -84 |
| 45 | 144 |  | 109 | 43 |  | 401 | 77 |  | 512 | 73 |  | 614 | -85 |
| 72 | 144 |  | 161 | 43 | -86 | 512 | 74 |  | 490 | 46 |  | 353 | -85 |
| 136 | 142 |  | 81 | 43 |  | 398 | 51 |  | 369 | 45 |  | 657 | -86 |
| 70 | 141 |  | 78 | 43 |  | 722 | 51 |  | 516 | 45 |  | 16 | -88 |
| 56 | 139 |  | 107 | 42 |  | 757 | 51 |  | 420 | 45 |  | 193 | -127 |
| 119 | 135 |  | 49 | 42 | -83 | 452 | 51 |  | 467 | 44 |  | 366 | -129 |
| 62 | 101 |  | 65 | 42 |  | 741 | 50 |  | 519 | 44 |  | 556 | -134 |
| 110 | 100 |  | 96 | 42 |  | 735 | 50 |  | 460 | 43 |  | 20 | -168 |
| 128 | 99 |  | 126 | 41 |  | 626 | 50 |  | 481 | 42 | -40 | 341 | -169 |
| 117 | 98 |  | 100 | 41 |  | 764 | 49 |  | 581 | 42 |  | 179 | -288 |
| 75 | 97 |  | 91 | 41 |  | 151 | 49 |  | 777 | 42 |  |  |  |
| 123 | 96 |  | 73 | 40 |  | 381 | 49 |  | 574 | 42 |  |  |  |
| 135 | 96 |  | 38 | 40 |  | 827 | 49 |  | 757 | 41 |  |  |  |
| 111 | 95 |  | 101 | 40 |  | 792 | 49 |  | 672 | 41 |  |  |  |
| 93 | 94 |  | 157 |  | -39 | 366 | 48 |  | 458 | 41 |  |  |  |
| 79 | 93 |  | 66 |  | -40 | 653 | 48 |  | 415 | 41 |  |  |  |
| 129 | 93 |  | 105 |  | -40 | 692 | 48 |  | 697 | 41 | -84 |  |  |
| 126 | 91 |  | 125 |  | -40 | 704 | 48 |  | 690 | 40 |  |  |  |
| 94 | 88 |  | 98 |  | -41 | 392 | 47 |  | 630 | 40 |  |  |  |
| 100 | 87 |  | 140 |  | -41 | 375 | 47 |  | 545 | 40 |  |  |  |
| 149 | 85 |  | 85 |  | -42 | 441 | 47 |  | 359 | 39 |  |  |  |
| 160 | 51 |  | 120 |  | -42 | 25 | 46 |  | 437 | 39 |  |  |  |
| 154 | 51 |  | 110 |  | -42 | 340 | 46 |  | 449 | 38 |  |  |  |
| 83 | 51 |  | 111 |  | -42 | 446 | 46 |  | 34 | 37 |  |  |  |
| 91 | 51 |  | 152 |  | -42 | 682 | 46 |  | 358 | 37 |  |  |  |
| 120 | 50 |  | 64 |  | -43 | 694 | 45 |  | 511 | 37 |  |  |  |
| 99 | 49 |  | 158 |  | -45 | 606 | 45 |  | 183 | 36 |  |  |  |
| 144 | 49 |  | 75 |  | -46 | 463 | 45 |  | 623 | 36 |  |  |  |
| 24 | 49 |  | 45 |  | -47 | 339 | 45 |  | 567 |  | -35 |  |  |
| 121 | 48 |  | 127 |  | -81 | 701 | 45 |  | 583 |  | -35 |  |  |
| 77 | 48 |  | 123 |  | -84 | 594 | 43 |  | 258 |  | -36 |  |  |
| 107 | 48 |  | 80 |  | -85 | 674 | 42 |  | 186 |  | -37 |  |  |
| 122 | 47 |  | 133 |  | -86 | 146 | 42 |  | 417 |  | -37 |  |  |
| 80 | 47 |  | 151 |  | -91 | 335 | 42 |  | 582 |  | -38 |  |  |
| 118 | 47 |  | 117 |  | -131 | 153 | 42 |  | 554 |  | -38 |  |  |
| 106 | 46 |  | 164 |  | -168 | 785 | 42 |  | 606 |  | -39 |  |  |
| 108 | 46 |  | 121 |  | -173 | 686 | 42 |  | 536 |  | -39 |  |  |
| 147 | 46 |  | 156 |  | -220 | 24 | 41 |  | 300 |  | -39 |  |  |
| 85 | 45 |  | 163 |  | -246 | 649 | 40 |  | 594 |  | -39 |  |  |
| 68 | 44 |  | 142 |  | -254 | 338 | 40 |  | 799 |  | -39 |  |  |
| 109 | 43 |  | 135 |  | -269 | 697 | 37 |  | 444 |  | -40 |  |  |
| 52 | 43 |  | 95 |  | -308 | 187 | 37 |  | 731 |  | -40 |  |  |
| 82 | 42 |  |  |  |  | 517 |  | -44 | 144 |  | -41 |  |  |
| 43 | 38 |  |  |  |  |  |  |  | 19 |  | -41 |  |  |
| 124 |  | -43 |  |  |  |  |  |  | 570 |  | -41 |  |  |
| 130 |  | -44 |  |  |  |  |  |  | 601 |  | -41 |  |  |
| 132 |  | -47 |  |  |  |  |  |  | 393 |  | -41 |  |  |
| 155 |  | -51 |  |  |  |  |  |  | 352 |  | -42 |  |  |
| 34 |  | -51 |  |  |  |  |  |  | 13 |  | -42 |  |  |
| 71 |  | -102 |  |  |  |  |  |  | 381 |  | -42 |  |  |

**Note**: This table is based on the antiTB dataset. If a feature exists (e.g. bit137=1), then sign = 1, otherwise (bit 137=0) sign = −1. Rank in Active means the rank of a feature in active compounds and Rank in Inactive for a feature in inactive compounds. The rank value is computed by equation 1. For Bit 137, it means both bit137=1 and bit137=0 are discovered in the rules for inactives. The rank for bit137=1 and bit137=0 for inactives is 44 and 83 respectively. Yellow features only exist in active compounds; red only in inactive compounds; green in both types.

**Table S2** **Important MDL features for the antiTB dataset**

| **Only exist in active compounds** | | | | | |
| --- | --- | --- | --- | --- | --- |
| 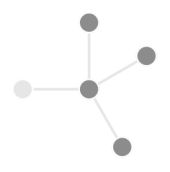 | 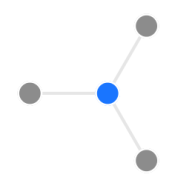 | Heterocyclic atom > 1 | 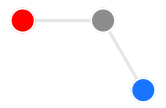 | 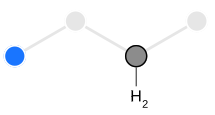 | |
| 66 CC(C)(C)A | 85 CN(C)C | 120 | 110 NCO | 111 NACH2A | |
| 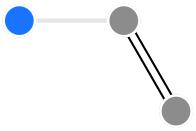 | 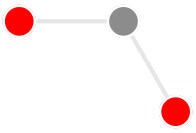 | 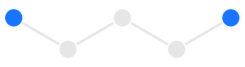 | 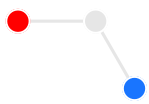 | 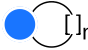 | |
| 45 C=CN | 123 OCO | 80 NAAAN | 117[#7]~*~[#8] | 121[#7;R] | |
| 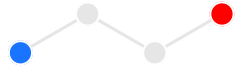 | 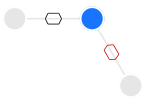 | 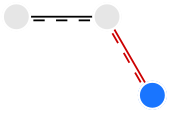 |  |  | |
| 95[#7]~*~*~[#8] | 75 | 135[#7]!:*:* |  |  | |
| Only exist in inactive compounds | | | | | |
| 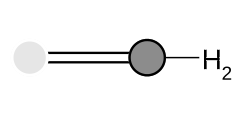 |  |  |  |  | |
| 34 CH2=A |  |  |  |  | |
| Exist in both active and inactive compounds | | | | | |
| 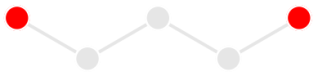 | 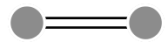 | 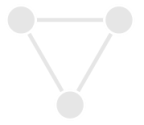 | 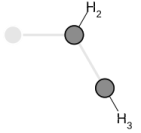 | | 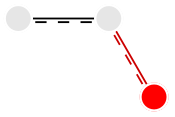 |
| 89[#8]~*~*~*~[#8] | 99[#6]=[#6] | 22*1~*~*~1 | 114[CH3]~[CH2]~* | | 113[#8]!:*:* |

**Note**: Each bit corresponds to a SMARTS pattern [48] which consists of two fundamental types of symbols: atoms and bonds. “*” means any atom, “A” an aliphatic atom, “~” any bond and “:” aromatic bond. So Bit 89, [#8]~*~*~*~[#8], means “two oxygen atoms connected by three unspecified atoms with any type of bonds”.

**Table S3** **Important PubChem features for the antiTB dataset**

| **Only exist in active compounds** | | | |
| --- | --- | --- | --- |
| 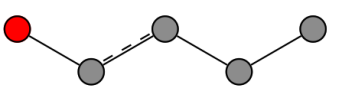 | 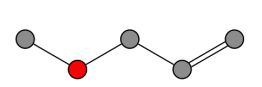 | 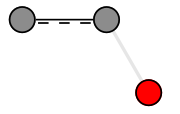 | 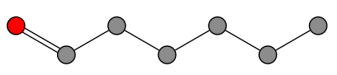 |
| 606 O-C:C-C-C | 594 C-O-C-C=C | 381 C(~O)(:C) | 692 O=C-C-C-C-C-C |
| 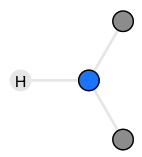 | 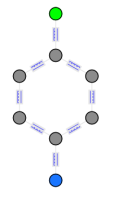 | 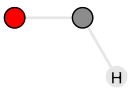 |  |
| 392 N(~C)(~C)(~H) | 792 NC1CCC(Cl)CC1 | 366 C(~H)(~O) |  |
| Exist in both active and inactive compounds | | | |
| >= 5 saturated or aromatic carbon-only ring size 6 | >= 1 saturated or aromatic carbon-only ring size 3 | 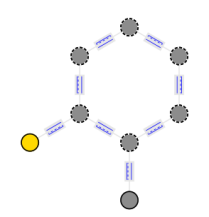 | >= 1 unsaturated non-aromatic nitrogen-containing ring size 6 |
| 207 | 116 | 757 Cc1c(S)cccc1 | 183 |

**Table S4** **Related features among top 10 of MDL and PubChem fingerprints**

| **MDL** | **PubChem** |
| --- | --- |
| Active | |
| 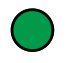 | 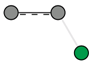 |
| 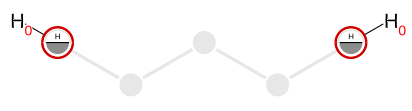 | 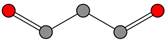 |
| 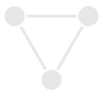 | >= 1 saturated or aromatic carbon-only ring size 3 |
| 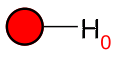 | 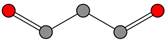 |
| Inactive | |
| 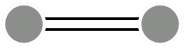 | 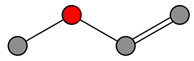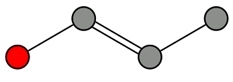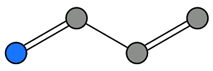 |
| 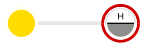 | 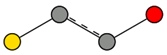 |

**Note**: All visualized SMARTS patterns are generated by using smartsviewer from http://smartsview.zbh.uni-hamburg.de/. The color scheme uses the popular CPK coloring with green for fluorine, red for oxygen, black for carbon, yellow for sulfur and blue for nitrogen.

**Table S5** **The matched molecules for rule 1**–**4 in table** **7**

| a. Rule 1 | b. Rule 2 |
| --- | --- |
| 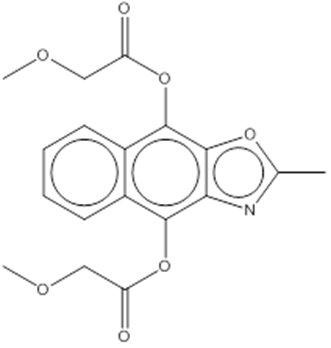 | 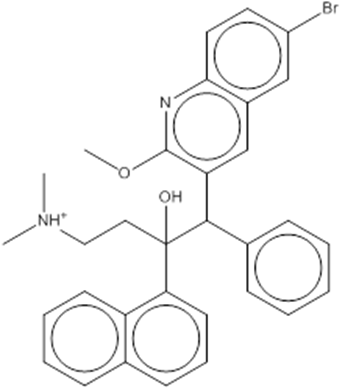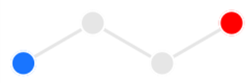 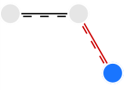 |
| c. Rule 3 | d. Rule 4 |
| 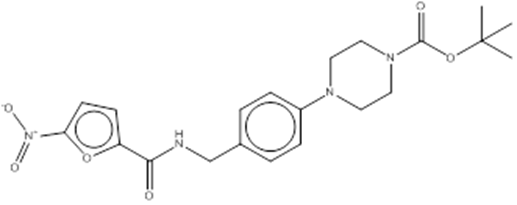 | 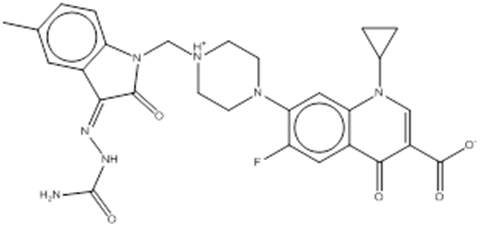 |

**Note**: **a**. red shape is *!@[#8]!@* and green shape [#7]~[#6]~[#8] **b**. molecule does not contain the two substructures **c**. red shape is *~*(~*)(~*)~* and green shape is [#7]~[#6]~[#8] **d**. red shape is [#7]~*~[CH2]~* and green shape is [#8]~[#6]~[#8]
